# MiRNA-93: a novel signature in human disorders and drug resistance

**DOI:** 10.1186/s12964-023-01106-3

**Published:** 2023-04-19

**Authors:** Bashdar Mahmud Hussen, Snur Rasool Abdullah, Mohammed Fatih Rasul, Zanko Hassan Jawhar, Goran Sedeeq Hama Faraj, Arda Kiani, Mohammad Taheri

**Affiliations:** 1 Department of Clinical Analysis, College of Pharmacy, Hawler Medical University, Kurdistan Region, Erbil, Iraq; 2 Medical Laboratory Science, College of Health Sciences, Lebanese French University, Kurdistan Region, Erbil, Iraq; 3 Department of Pharmaceutical Basic Science, Faculty of Pharmacy, Tishk International University, Erbil, Kurdistan Region Iraq; 4 Department of Medical Laboratory Science, Komar University of Science and Technology, Sulaymaniyah, Iraq; 5 Loghman Hakim Hospital, Shahid Beheshti University of Medical Sciences, Tehran, Iran; 6 Institute of Human Genetics, Jena University Hospital, Jena, Germany; 7 Urology and Nephrology Research Center, Shahid Beheshti University of Medical Sciences, Tehran, Iran

**Keywords:** Cancer, miRNA-93, Biomarker, Drug resistance

## Abstract

miRNA-93 is a member of the miR-106b-25 family and is encoded by a gene on chromosome 7q22.1. They play a role in the etiology of various diseases, including cancer, Parkinson’s disease, hepatic injury, osteoarthritis, acute myocardial infarction, atherosclerosis, rheumatoid arthritis, and chronic kidney disease. Different studies have found that this miRNA has opposing roles in the context of cancer. Recently, miRNA-93 has been downregulated in breast cancer, gastric cancer, colorectal cancer, pancreatic cancer, bladder cancer, cervical cancer, and renal cancer. However, miRNA-93 is up-regulated in a wide variety of malignancies, such as lung, colorectal, glioma, prostate, osteosarcoma, and hepatocellular carcinoma. The aim of the current review is to provide an overview of miRNA-93's function in cancer disorder progression and non-cancer disorders, with a focus on dysregulated signaling pathways. We also give an overview of this miRNA's function as a biomarker of prognosis in cancer and emphasize how it contributes to drug resistance based on in vivo, in vitro, and human studies.

Video Abstract

**Supplementary Information:**

The online version contains supplementary material available at 10.1186/s12964-023-01106-3.

## Introduction

MicroRNAs (miRNAs) are small RNA molecules that regulate gene expression post-transcriptionally by acting on the stability and translation of transcribed mRNAs [1]. The RNA polymerase II enzyme generates polyadenylated and cap-coated pri-miRNAs [2]. This transcript is further processed by Drosha and Dicer ribonuclease to generate stem-loop precursor miRNA and mature miRNA [3]. Even this component is part of an RNA-induced silencing complex that can recognize certain mRNA targets, therefore preventing translation or making the mRNA unstable [4]. MiRNAs play regulatory roles in cancer, such as altering the signaling axis [5], modulation of gene expression [6], and biological processes like cell growth, division, apoptosis, and maintaining homeostasis [7], all of which suggest that they participate in the etiology of illness.

MiRNA-93 is encoded by a gene on chromosome 7q22.1 [8]. They are expressed in the nucleus and co-transcribed with the host minichromosome maintenance complex component 7 (*MCM7*) gene [9]. It is a paralog of the miRNA-17–92 cluster [10], a member of the pro-oncogenic miRNA-106b-25 cluster. This cluster has been shown to regulate the expression of various target genes involved in important cellular processes such as cell proliferation, apoptosis, and angiogenesis [10, 11].

Studies have shown that miRNA-93 is upregulated in several types of cancer, such as breast cancer (BC) [12], lung cancer [13], colorectal cancer [14], prostate cancer [15], and pancreatic cancer [16]. In these cancers, miRNA-93 acts as oncogenic miRNA and promotes tumor growth and metastasis through regulating the expression of targeted genes involved in cell proliferation, angiogenesis, and invasion [17, 18]. For example, Fang and his colleagues revealed that miRNA-93 might stimulate both tumor development and angiogenesis via inhibiting the expression of integrin-8 [19].

In contrast, miRNA-93 can inhibit tumor growth in a number of ways. For instance, in BC cells, overexpression of miR-93 reduced the protein level of Wiskott-Aldrich syndrome protein family member 3 (*WASF3*), a regulator of CSC characteristics and cytoskeleton remodeling, and *WASF3* reversed the miRNA-93-mediated reduction of BC cell invasion [20]. These results contribute to the role of miRNA-93 as a metastasis inhibitor by reducing BC's invasiveness and stem cell characteristics.

In addition to cancer, miRNA-93 has also been implicated in non-malignant disorders, such as osteoarthritis [21], rheumatoid arthritis (RA) [22], atherosclerosis [23], hepatic injury [24], Parkinson's disease [25], acute myocardial infarction [26], and chronic kidney disease [27]. Matrix metalloproteinase 3 (MMP3), a proteolytic enzyme that breaks down collagen fiber that is primarily produced in inflamed joints and ultimately plays a substantial role in joint inflammation and bone erosion in RA, is a key participant in the etiology of the disease [28]. It was discovered that *MMP3* is a potential miRNA-93 target. By specifically targeting the 3′UTR of *MMP3*, miRNA-93 reduced *MMP3* expression [29]. Furthermore, the methylation of the promoter *MMP3* gene is caused by the overexpression of the miRNA-93 gene [22].

Recently, miRNA-93 participates in drug resistance of tumor cells by targeting on genes or signaling pathways linked to resistance development, such as the PI3K/Akt signaling pathway and the expression of anti-apoptotic proteins in pancreatic cancer [30]. Moreover, miRNA-93, a potential *PTEN/Akt* signaling pathway inhibitor, controls the chemosensitivity of ovarian cancer cells to the chemotherapy agent cisplatin [31]. Further, miRNA-93 may play an important role in EMT and drug resistance of BC cells by targeting *PTEN*. For example, Chu et al. identified that miRNA-93 targets *PTEN* to help BC cells undergo EMT and acquire doxorubicin resistance [32].

The current study attempts to review miRNA-93's involvement in malignant and non-malignant diseases, focusing on its target mRNA and dysfunctional signaling cascades, as well as its function in drug resistance and cancer prognosis.

## Search methodology

Based on the primary keywords (miRNA-93, malignant disorders, non-malignant disorders, drug resistance), we conducted searches on PubMed, a well-known database of biological literature. The real samples were included in accordance with the parameters of the review study on miRNA-93 related to the malignant and non-malignant disorders and drug resistance. Conversely, the study did not include the disqualifying data.

## miRNA-93 expression in malignant conditions

Numerous miRNAs are affected by cancer, and depending on the situation, they might function as tumor suppressors or oncogenes. Recent studies in cancer cell lines, animal models of cancer, and cancer patient samples have highlighted the role of miRNA-93 in carcinogenesis. Utilizing these three datasets, we proceed to characterize miRNA-93's function in carcinogenesis in the following sections.

### Cell line studies

#### Up-regulation of miRNA-93 in cancer cell lines

Different kinds of cell lines were used in vitro in order to find how miRNA-93 upregulation induces cancer progression **(**Table 2**)**. For example, miRNA-93 significantly increased in TNBC by 6.92-fold and has been linked to TNM grade, Ki-67 staining, and lymph node metastases in TNBC patients when compared to non-TNBC tissues or normal tissues [33]. Shyamasundar et al. revealed that miRNA-93 was found to be increased by 60-fold and prevents the invasive potential of TNBC cells through the protein kinase WNK1 [34]. Likewise, Feng and his colleagues showed that in vascular smooth muscle cells (VSMCs), miRNA-93 is highly elevated in VSMCs in vivo and in vitro studies [35]. Their findings suggest that, via suppressing mitofusin 2 (*Mfn2*) expression, miRNA-93 promotes VSMC proliferation and migration.

**Fig. 1:**
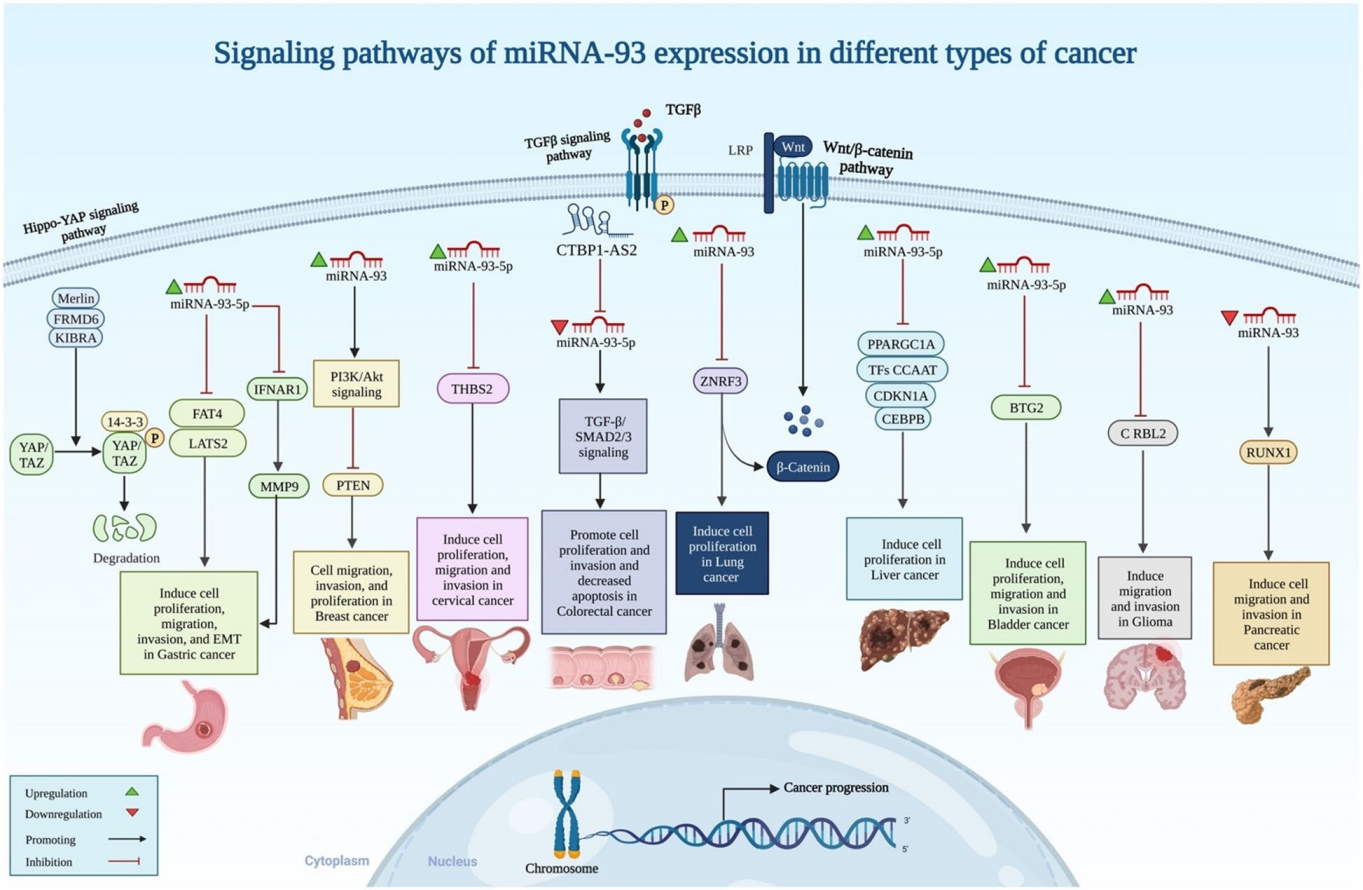
The expression patterns and pivotal roles of miRNA-93 in tumor networks are graphically depicted, along with its signaling pathways in different types of cancer

Furthermore, recent studies found that for the treatment of in-stent restenosis, miRNA-93 might be a potential target. For example, after controlling the standard risk factors (RFs), miRNA-93-5p was able to distinguish between individuals with stable coronary artery disease (CAD) and those without CAD [36]. Similarly, Feng et al. showed that *Mfn2* is a direct target of miRNA-93, which encourages VSMC migration and proliferation. They showed that for the treatment of in-stent restenosis, miRNA-93 may be a novel target [37]. Additionally, miRNA-93 was found to be greatly enhanced in an in vitro investigation by Xio et al., promoting cell migration and proliferation, while miRNA-93 repression had the reverse effect [38]. They revealed that miRNA-93-3p increased significantly by 2.5-fold and inhibits ZFP36 Ring Finger Protein Like 1 (*ZFP36L1*), which then induces Zinc Finger Protein X-Linked (*ZFX*) expression and stimulates keratinocyte migration and proliferation during skin wound healing. Moreover, Li et al. proved that targeting the Phosphatase and tensin homolog (*PTEN*) gene with miRNA-93 controls the *PTEN/PI3K/Akt* pathway in BC cells, which increases tumor cell proliferation, invasion, and migration in vitro [12] (Fig. 1). Based on their data, *PTEN* is directly targeted by this miRNA and could act as a promising therapeutic target for BC.

According to these studies, miRNA-93 has been upregulated in cancer cell lines and promoted the progression of several kinds of cancer.

#### Down-regulation of miRNA-93 in cancer cell lines

MiRNA-93 is often upregulated in cancer cell lines; however, it can also be downregulated in other cancer cell lines. For instance, in colorectal cancer (CRC), miRNA-93 has anti-tumor properties such as reducing colon cancer cell motility, proliferation, and angiogenesis [39]. CTBP1-AS2 was shown to be overexpressed in colorectal cancer by Li et al., and it enhances the activation of the *TGF-/SMAD2/3* signaling pathway by blocking miRNA-93-5p, which in turn allows rapid progression of CRC [40]. Furthermore, Qu et al. approved that miRNA-93 in lung cancer (LC) cells is overexpressed and directly attach to the 3′-UTR of the Neural precursor cell expressed developmentally downregulated gene 4-like (*NEDD4L*) messenger RNA (mRNA), which leads to the production of NEDD4L to be downregulated at the protein level and promoted TGF-β induced EMT [41]. Additionally, Xiang and his team demonstrated that in BC cells, megakaryoblastic leukemia 1 (*MKL-1*) gene and signal transducer and activator of transcription 3 (*STAT3*) expression are both suppressed by miRNA-93-5p through targeting their 3'UTR, which prevents BC cells from undergoing EMT [42]. They approved the fact that miRNA-93-5p regulates *MKL-1* and *STAT3*, which influence the EMT process, and that it can control BC cell migration. These findings suggest that miRNA-93 is expressed differently in different cancer cell lines. The functions of miRNA-93 in a number of cancer cell lines are summarized in Table 1.

**Table 1:** In various cancer cell lines, the functions of miRNA-93 and important targets with associated phenotypes are highlighted

Tumor type	miRNAs	Levels incancer cell lines compared with normal cell lines	Interactions	Downstream target of miRNA	Effect of miRNA-93 up-regulation on its target	Cell line	Associated phenotypes with dysregulation of miRNA-93	Ref
Lung Cancer	miRNA-93	Up	FUS1	FUS1	Inhibition	NCI-H146, NCI-H157, NCI-H187, NCI-H209, NCI-H526, NCI-H889, NCIH1299, NCIH1648, NCI-H1672, NCI-H1770, NCI-H1819, NCI-H2052, NCI-H2107, NCI-H2171, NCI-H2195, NCI-H2122, NCI-H2887, HCC366, HCC970, HCC1195, HCC2450	↑ miRNA-93, ↓ FUS1: ↑ tumor progression	[43]
	miRNA-93-5p	Up	PTEN/RB1	PTEN/RB1	Inhibition	A549, SK-MES-1	↑ miRNA-93-5p, ↓ PTEN/RB1: ↑ Cell proliferation, migration, and invasion	[44]
	miRNA-93	Up	NEDD4L	NEDD4L	Inhibition	A549, H1650	↑ miRNA-93, ↓ NEDD4L: ↑ tumorigenesis and metastasis	[41]
	miRNA-93	Up	DAB2	DAB2	Inhibition	H1993	↑ miRNA-93, ↓ DAB2: ↑ tumor growth	[45]
	miRNA-93	Up	PI3K/Akt, LKB1, PTEN, p21, CDKN1A	LKB1/PTEN /CDKN1A	Inhibition	A549, NCI-H1975, NCI-H1299	↑ miRNA-93, ↓ LKB1/PTEN /CDKN1A/p21, ↑ PI3K/Akt: ↑ tumorigenesis and metastasis	[46]
	miRNA-93	Up	ZNRF3, Wnt/β-catenin	ZNRF3	Inhibition	A549, H460	↑ miRNA-93, ↓ZNRF3, ↓ Wnt/β-catenin: ↑ cell proliferation	[47]
Breast Cancer	miRNA-93-5p	Up	MKL-1 /STAT3	MKL-1 /STAT3	Inhibition	MCF-7, MDA-MB-231, T47D	↑ miRNA-93-5p, ↓ MKL-1 /STAT3: ↑ migration, ↓EMT	[42]
	miRNA-93	Up	PI3K/Akt, PTEN	PTEN	Inhibition	MDA-MB-231	↑ miRNA-93, ↓ PTEN, ↑ PI3K/Akt: ↑ cell migration, invasion, and proliferation	[12]
	miRNA-93	Down	WNK1	WNK1	Inhibition	MDA-MB-231	↑miRNA-93, ↓ WNK1: ↓ invasive	[34]
	miRNA-93	Downregulated (by lncRNA-H19)	STAT3, lncRNA-H19	STAT3	Inhibition	HEK293T, MCF‐7, MDA‐MB‐231	↑ lncRNA-H19, ↓ miRNA-93, ↑ STAT3: ↓proliferation	[48]
	miRNA-93	Up	LATS2	LATS2	Inhibition	MT-1	↑ miRNA-93-5p, ↓ LATS2: ↑ angiogenesis and metastasis	[49]
Colorectal Cancer	miRNA-93-5p	Up	MDR1, CDKN1A	CDKN1A	Inhibition	HCT‑8, MDR HCT‑8/vincristine (VCR)	↑ miRNA-93-5p, ↓CDKN1A, ↑ MDR1: ↑ MDR	[14]
	miRNA-93-5p	Up	FOXA1, TGFB3	FOXA1	Inhibition	HT-29, SW480, LoVo	↑ miRNA-93-5p, ↓ FOXA1: ↑ tumor growth	[50]
	miRNA-93	Down	Smad7, Wnt/β-catenin	Smad7	Inhibition	HCT116, HT29, SW480, SW620, LoVo, LS174T	↓ miRNA-93, ↑ Wnt/β-catenin, ↓ Smad7: ↓ tumor growth	[51]
	miRNA-93-5p	Downregulated (by CTBP1-AS2)	TGF-β/SMAD2/3, CTBP1-AS2	TGF-β	Inhibition	Caco-2, SW620, HT29, T84, HCT116, SW480	↑ CTBP1-AS2, ↓miRNA-93-5p, ↑ TGF-β/SMAD2/3 pathway: ↑ cell proliferation and invasion and decreased apoptosis	[40]
	miRNA-93	Up	CCNB1, ERBB2, P21, VEGF	CCNB1, ERBB2, P21, VEGF	Inhibition	Caco2, LoVo, HCT116 (ATCC, Manassas, VA, USA)	↑ miRNA-93-5p, ↓ CCNB1, ERBB2, P21, VEGF: ↓ tumorigenesis	[39]
	miRNA‐93‐5p	Down	MMP‐1, 2, MMP-9, IL‐2, IFN‐γ, TNF‐α, PD‐L1	PD‐L1	Inhibition	HCT116, SW480	↓ miRNA-93-5p, ↑ PD‐L1, ↓ MMP‐1, 2, MMP‐9: ↓ migration and invasion	[52]
	miRNA-93	Downregulated by lncRNA CA3-AS1	lncRNA CA3-AS1, PTEN	PTEN	Inhibition	HCT-116, SW480, SW620, SW1116, HT29	↑ CA3-AS1, ↓ miRNA-93, ↑ PTEN: ↑ apoptosis, ↓ proliferation, invasion	[53]
Liver Cancer	miRNA-93	Up	TIMP2, TP53INP1, CDKN1A	TIMP2/TP53INP1 /CDKN1A	Inhibition	WRL68, HepG2, SMMC7721, SKHEP1, HUH7	↑ miRNA-93, ↓ TIMP2, TP53INP1, CDKN1A: ↑ proliferation and invasion	[54]
	miRNA-93	Up	PDCD4	PDCD4	Inhibition	HEK293T, SMMCC-7721, Huh-7	↑ miRNA-93, ↓ PDCD4: ↑migration and invasion	[55]
	miRNA-93	Up	T-ICs, MTMR3	MTMR3	Inhibition	HCCLM3, EpCAM + , EpCAM −	↑ miRNA-93, ↓ MTMR3, ↑T-ICs: ↑ resistance to sorafenib treatment	[56]
	miRNA-93-5p	Up	PPARGC1A, CDKN1A, CEBPB, TFs CCAAT	PPARGC1A, CDKN1A, CEBPB, TFs CCAAT	Inhibition	293 T, L-02, a SMMC-7721, Huh-7, SK-Hep-1, HepG2, HCCLM3, and MHCC97H	↑ miRNA-93-5p, ↓ PPARGC1A, CDKN1A, CEBPB, TFs CCAAT: ↑cell proliferation	[57]
	miRNA-93	Up	PDCD4	PDCD4	Inhibition	QGY-7703, SMMC-7721	↑ miRNA-93, ↓ PDCD4: ↑ cell proliferation	[58]
	miRNA-93-5p	Up	MAP3K2, MKK4, P38, JNK, c-Jun	-	-	HepG2, BEL-7402, Hep3B, MHCC-97L, MHCC-97H, HCC-LM3	↑ miRNA-93-5p, ↑ MAP3K2, MKK4, P38, JNK, c-Jun: ↑ proliferation	[59]
	miRNA-93	Up	PTEN, CDKN1A, c-Met/PI3K/Akt	PTEN, CDKN1A	Inhibition	HepG2, Hep3B, PLC/PRF/5, SNU398, SNU423, SNU449	↑ miRNA-93, ↓ PTEN, CDKN1A, ↑ c-Met/PI3K/Akt: ↑ cell proliferation, migration, and invasion	[60]
Gastric Cancer	miRNA-93-5p	Up	IFNAR1, MMP9, STAT3	IFNAR1	Inhibition	MGC803, MKN28, SGC-7901, HGC-27, BGC-823, MKN45, AGS, HEK293	↑ miRNA-93-5p, ↓IFNAR1, ↑ STAT3: ↑cell metastasis	[61]
	miRNA-93	Up	PDCD4	PDCD4	Inhibition	AGS	↑ miRNA-93-5p, ↓ PDCD4: ↑ tumor growth	[62]
	miRNA-93-5p	Up	FAT4, LATS2	FAT4, LATS2	Inhibition	SGC-7901, HGC-27	↑ miRNA-93-5p, ↓FAT4, LATS2: ↑ tumor progression	[63]
	miRNA-93	Up	TIMP2	TIMP2	Inhibition	SGC-7901, MKN-28, BGC-823, MGC-803, MKN-45, GES-1	↑ miRNA-93, ↓ TIMP2: ↑ proliferation and metastasis	[64]
	miRNA-93-5p	Up	AHNAK, DKK1	AHNAK	Inhibition	SUN-216, BGC-823, MKN74, HGC-27, GES-1	↑ miRNA-93-5p, ↓ AHNAK: ↑cell migration, invasion and EMT	[65]
	miRNA-93-5p	Downregulated (by Matrine)	AHNAK	AHNAK	Inhibition	MKN‐28, SGC‐7901	↑ Matrine, ↓ miRNA-93-5p, ↑ AHNAK: ↑proliferation, migration and invasion	[66]
Pancreatic Cancer	miRNA-93	Up	CRMP-2, YES1, MAPRE1	CRMP-2, YES1, MAPRE1	Inhibition	HPDE, HEK-293 T, PANC-1, MIA PaCa-2	↑ miRNA-93, ↓ CRMP-2, YES1, MAPRE1: ↑tumor progression	[67]
	miRNA-93	Down	RUNX1, HMGA2	RUNX1	Inhibition	PANC-1, MIA PaCa-2	↓ miRNA-93, ↑RUNX1: ↑cell migration and invasion	[68]
Bladder Cancer	miRNA-93-5p	Up	BTG2	BTG2	Inhibition	T24, UM-UC-3, SV-HUC-1	↑ miRNA-93-5p, ↓ BTG2: ↑ proliferation, migration and invasion	[69]
	miRNA-93	Up	PEDF	PEDF	Inhibition	TCCSUP, 5637, UM-UC-3, T24	↑ miRNA-93, ↓ PEDF: ↑ proliferation and invasion	[70]
	miRNA-93-5p	Up	KLF9	KLF9	Inhibition	SV-HUC-1, 5637, RT-112, RT4, BT-B	↑ miRNA-93-5p, ↓ KLF9: ↑ proliferation and migration	[71]
	miRNA-93	Down	LASS2	LASS2	Inhibition	RT4, T24	↓ miRNA-93, ↑ LASS2: ↑ chemo-sensitivity	[72]
Cervical Cancer	miRNA-93-5p	Up	THBS2, MMPS	THBS2	Inhibition	siHa, 293 T	↑ miRNA-93-5p, ↓ THBS2: ↑ proliferation, invasion and migration	[73]
	miRNA-93-5p	Up	BTG3, HR‑HPV	BTG3	Inhibition	SiHa, CaSk, HeLa, C4-1, C33A	↑ miRNA-93-5p, ↓BTG3: ↓ proliferation, invasion and migration	[74]
	miRNA-93	Up	CDKN1A	CDKN1A	inhibition	Hela	↑ miRNA-93, ↓ CDKN1A: ↑proliferation and invasion	[75]
	miRNA-93	Downregulated (by MCM3AP-AS1)	-	-	-	C-33A (HPV-negative) and SiHa (HPV positive)	↑ MCM3AP-AS1, ↓miRNA-93: ↓ cell proliferation	[76]
Prostate Cancer	miRNA-93	Up	TGFΒR2, ITGB8, LATS2	-	-	RWPE-1, PC-3, LNCaP, DU145, 22RV1	↑ miRNA-93, ↑ TGFΒR2, ITGB8, LATS2: ↑proliferation and invasion	[15]
	miRNA-93	Up	DAB2, Akt/ERK1/2	DAB2	Inhibition	PC-3, DU145	↑ miRNA-93-5p, ↓ DAB2: ↑ progression and metastasis	[77]
Glioma	miRNA-93	Up	PTEN, PHLPP2, FOXO3, PI3K/Akt	PTEN, PHLPP2, FOXO3	Inhibition	U251MG, A172, LN229, SF767, U118MG, U87MG, Hs683, LN18, SHG44	↑ miRNA-93, ↓ PTEN, PHLPP2, FOXO3, C PI3K/Akt signaling: ↑ Proliferation	[78]
	miRNA-93	Up	P21	P21	Inhibition	U87, U251, SF126, SF767, A172, SHG44	↑ miRNA-93, ↓ P21: ↑ proliferation, colony formation, drug resistance	[79]
	miRNA-93	Up	RBL2	RBL2	Inhibition	NHAs, U251, U87	↑ miRNA-93, ↓ C RBL2: ↑ migration and invasion	[80]
	miRNA-93-5p	Down	MMP2	MMP2	Inhibition	U87-MG, LN-18	↓ miRNA-93, ↑MMP2: ↓ proliferation and metastasis	[81]
	miRNA-93	Up	IL-8, VEGF	IL-8, VEGF	Inhibition	U251, T98G	↑ miRNA-93, ↓ IL-8, VEGF: ↑angiogenesis	[82]
Osteosarcoma	miRNA-93	Up	PTEN, Akt	PTEN	Inhibition	HOS, SaOS, MG-63, NY, Hu09,	↑ miRNA-93, ↓ PTEN: ↑proliferation	[83]
	miRNA-93	Up	P21	P21	Inhibition	Saos-2, U2OS, SW1353, MG63, hFOB1.19, HEK293	↑ miRNA-93, ↓ P21: ↑ proliferation	[84]
	miRNA-93	Up	TIMP2, MMPs	TIMP2	Inhibition	U-2OS, OS-732, HOS, Saos-2, hFOB	↑ miRNA-93, ↓ TIMP2: ↑ cell viability, invasion, and EMT	[85]
Renal cancer	miRNA-93	Down	RBL2, TGF-beta	RBL2	Inhibition	786-O, 796-P	↓ miRNA-93, ↑ RBL2: ↑ tumor progression	[86]
Esophageal carcinoma	miRNA-93-5p	Up	TGFβR2	TGFβR2	Inhibition	Het-1A (BNCC337688), and EC cell lines [TE-1 (BNCC100151), Eca-109 (BNCC337687) and EC9706 (BNCC339892)]	↑ miRNA-93-5p, ↓ TGFβR2: ↑ proliferation, migration and invasion, ↓ apoptosis	[87]
	miRNA-93-5p	Up	PTEN, p21, cyclin D1	PTEN	Inhibition	EC9706	↑ miRNA-93-5p, ↓ PTEN, p21, ↑ cyclin D1: ↑ proliferation	[88]
Papillary thyroid carcinoma	miRNA-93-3p	Downregulated (by ASMTL-AS1)	ASMTL-AS1, miR-93-3p, miR-660, FOXO1	FOXO1	Inhibition	Nthy-ori 3–1	↑ ASMTL-AS1, ↓ miRNA-93-3p, miR-660, ↑ FOXO1: ↑ tumor growth and glycolysis	[89]

### Animal studies

**Fig. 2:**
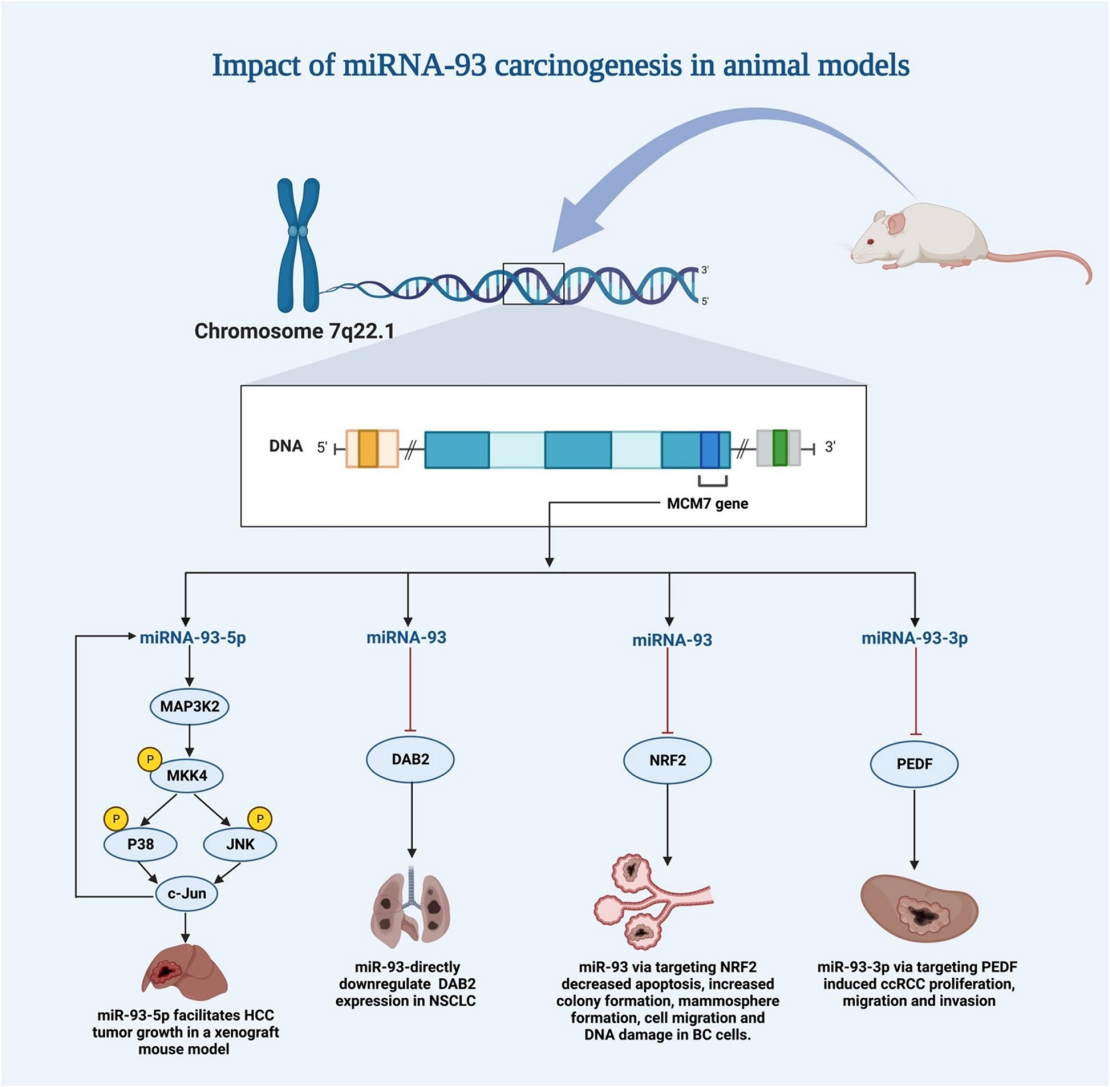
MiRNA-93's role in animal cancer models. By interacting with specific targets, miR-93-5p makes tumors grow rapidly in a xenograft mouse model of different types of cancer, such as, miR-93 directly inhibits the expression of *DAB2* in NSCLC. Targeting *PEDF* made RCC cells multiply and targeting *NRF2* inhibits cells from dying and made more colonies form in BC

MiRNA93 enhances tumor development and tumor cell survival, according to extensive in vivo and in vitro experiments. For instance, Du et al. demonstrated that in mouse tumor xenografts of NSCLC, miRNA-93 overexpression enhances tumor growth and is mediated through down-regulating expression (Fig. 2). However, altering the levels of miRNA-93 and *DAB2* has an impact on cell survival DAB2 protein [45]. They found that the *miRNA-93/DAB2* pathway plays a key role in controlling how lung cancer gets worse. This is clear from the fact that high miRNA-93 expression levels are linked to both low *DAB2* levels and poor patient survival. Similarly, CTBP1-AS2 was shown to promote proliferation and invasion of CRC cells in vitro and in vivo by sponging miRNA-93-5p and activating the *TGF-/SMAD2/3* pathway [40]. Moreover, in another study, Singh and his team found that nuclear factor erythroid 2–related factor 2 (*NRF2*) and *NRF2*-regulated genes' protein expression was reduced by ectopic miRNA-93 expression in rat mammary tissue. As a result, miRNA-93 prevented the development of mamospheres, colony formation, apoptosis, cell migration, and DNA damage in breast epithelial cells, while silencing miRNA-93 in these cells promoted these cancer-causing activities [90]. They approved that miRNA-93 has a carcinogenic potential during E2-induced breast carcinogenesis. Additionally, Chen et al. showed in their study that ras homolog family member C (*RhoC*) is a target of miRNA-93-5P, which may prevent the growth and advancement of EOC tumors. Although, they suggested that miRNA-93-5P has the capacity to inhibit the growth of ovarian cells [91]. This result shows that *RhoC* is being downregulated in tumor xenografts in vivo by miRNA-93-5P in order to prevent EOC aggression, which may offer a deeper understanding of the molecular pathways behind cancer aggression. However, according to these studies, miRNA-93 might be an important factor in the advancement of cancers in vivo. Based on research in animal models, Table 2 shows how miRNA-93 affects carcinogenesis, along with the genes it affects and how it affects the progression of cancer.

**Table 2:** Impact of miRNA-93 in carcinogenesis based on research in animal models

Type of tumor	Types of miRNAs	Animal model	Methods of Manipulation and Cellular Grafting	Phenotypes linked to miRNA-93 dysregulation	Ref
NSCLC	miRNA-93	Nude mice	Injecting miR-93- or control-transfected H1993 cells subcutaneously	↑miRNA-93 (which targets DAB2): ↓tumor growth	[45]
Esophageal carcinoma	miRNA-93	Nude mice	Injections of transfected SKGT4 cells (circ-21–93 or circ-scr) under the skin	↑miRNA-93: ↓xenografted tumor growth	[92]
Hepatocellular carcinoma	miRNA-93-5p	BALB/c nude mice	Subcutaneous injection of lentiviral overexpression of miR-93-5p in SK-Hep-1 cells + lentiviral antagonist of miR-93-5p in MHCC-97H cells	↑miRNA-93-5p (directly targets MAP3K2): ↑ cell proliferation	[59]
Mammary tumor	miRNA-93	ACI rats	Injecting 3 mg of E2 pellets into MCF-10A and T47D cells infected with a lentiviral vector subcutaneously	↑miRNA-93(targets NRF2): ↑Clonability, development of mamospheres, and migratory characteristics of MCF-10A cells, and ↓ apoptosis	[90]
Epithelial ovarian carcinoma	miRNA-93-5p	BALB/c nude mice	Injections of miR-93-5P or mock-transfected OVCAR3 cells under the skin	↓miRNA-93-5p (directly targets RhoC): ↓ proliferation, ↑G1 or S arrest and apoptosis	[91]
Renal carcinoma	miRNA-93-3p	BALB/c nude mice	Transfected 786-O cells were injected subcutaneously with either a control lentivirus or an anti-miR-93-3p lentivirus	↑miRNA-93-3p (directly targets PEDF): ↑cell tumorigenesis and metastasis	[93]

### Studies in clinical samples

**Fig. 3:**
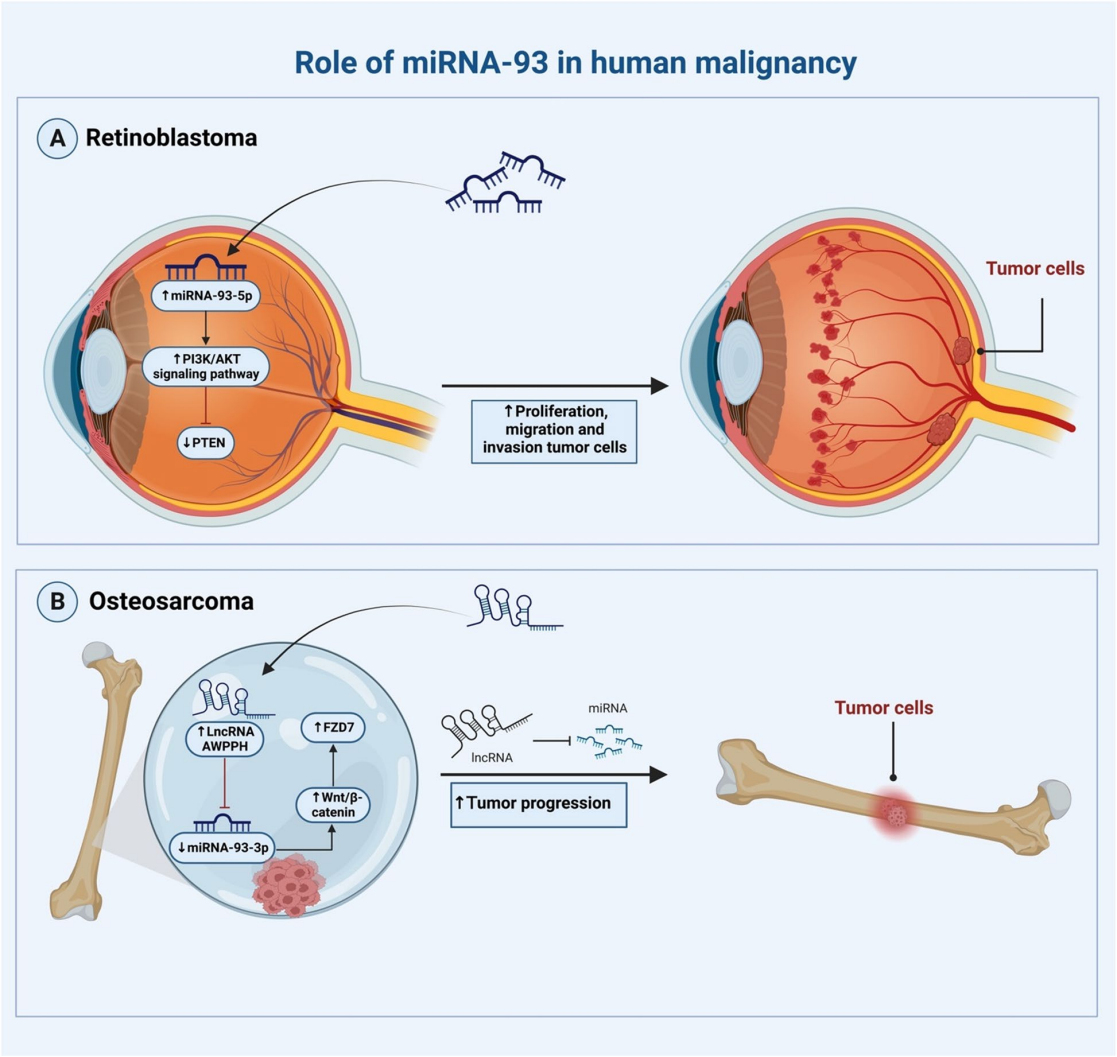
The function of miRNA-93 in human Retinoblastoma and Osteosarcoma. In a clinical sample of retinoblastoma and osteosarcoma, miRNA-93-5p interacts with particular targets to cause tumors to grow quickly. **A** Through the PI3K/AKT signaling pathway, miRNA-93 directly reduces the expression of *PTEN*, a tumor suppressor gene, which promotes the growth of tumors in RB. **B** The expression of *FZD7*, which promotes tumor growth, is stimulated by the Wnt/B-catenin pathway and the rising amount of lncRNA AWPPH, which sponges miRNA-93

Expression of miRNA-93 has been differentially expressed in different types of malignant tissues. Experimental studies in these tissues showed that changes in miRNA-93 expression are associated either positively or negatively with its target genes (Table 3). For instance, Xu et al., by using the qRT-PCR method for EC tissues and cells, found that the vitality and migration rate of EC cells were markedly boosted by miRNA-93-5p overexpression, which was responded to Interferon Alpha And Beta Receptor Subunit 1 (*IFNAR1*) up-regulation [94]. Likewise, miRNA-93-5p could accelerate the course of retinoblastoma by controlling apoptosis, cell proliferation, migration, and invasion in a way that is dependent on the *PTEN/PI3K/AKT* signaling cascade [95] (Fig. 3a). Similarly, Li and his team demonstrated that lncRNA AWPPH accelerates the development of osteosarcoma via modifying the *miR-93-3p/FZD7* axis, which activates the Wnt/b-catenin pathway [96] (Fig. 3b). Further, Xiao et al. showed that miRNA-93 targets cyclin G2 (*CCNG2*), which it in turn uses to increase proliferation, apoptosis inhibition, and increase migration and invasion of LSCC cells [97]. Moreover, according to Chen et al., miRNA-93-5p is likely increased by *CCND2* overexpression in ovarian cancer malignancy, which favors the growth and survival of ovarian cancer tumors [98]. Additionally, in SCCHN samples, miRNA-93-5p acts as an oncogene to inhibit repulsive guidance molecule BMP co-receptor b (*RGMB*), which in turn regulates invasion and migration [99]. These findings suggest that miRNA-93-5p may serve as a helpful biomarker for assessing the prognosis of cancer patients as well as a possible therapeutic target.

**Table 3:** miRNA-93 dysregulation in clinical samples and correlation between clinicopathologic traits and its expression in different types of cancer

Tumor/ disorder type	Samples	miRNA-93 expression (Tumor vs. Normal)	Cox regression and Kaplan–Meier analysis	Association of miRNA-93 expression with clinicopathologic characteristics	Ref
Breast carcinoma	20 BC patient tissue	miRNA-93 (Up)	-	Lymph node metastasis	[49]
Colon cancer	138 pairs of CC sample	miRNA-93 (Down)	Downregulation is associated with poor survival in patients	Positive nodal metastasis (*P* = 0.006), positive distant metastases (*P* = 0.01), and advanced tumor stage (*P* = 0.02)	[100]
Uterine cancer	176 UC sample + 100 healthy controls	miRNA-93 (Up)	The prognosis of UC patients is correlated with upregulation	Lymph node metastases and pathological stage	[8]
Laryngeal squamous cell carcinoma	59 pairs of LSCC samples	miRNA‐93‐5p (Up)	-	Histological grade, lymph node metastasis	[97]
Colorectal cancer	-First cohort: (35 non-early relapse CRC patients + 42 early relapse CRC patients) -Second cohort 45 CRC patients	miRNA-93 (Up)	An early UICC stage of CRC is linked to upregulation	G2 phase cell cycle arrest	[39]
Endometrial carcinoma	50 paired of EC tissues	miRNA‐93‐5p (Up)	Upregulation is associated with patients' low survival rates from EC	The EC's FIGO stage and lymph node metastases	[94]
Lacrimal gland adenoid cystic carcinoma	5 ACC patient tissues + 3 healthy controls	miRNA‐93‐5p (Up	-	Tumor migration, invasion, and proliferation	[101]
Ovarian cancer	-	miRNA-93-5p (Down)	Downregulation is linked to a poor prediction of patient survival time	Weakly increase OC cell apoptosis and inhibit cell migration	[98]
Esophageal cancer	30 ESCA samples + 30 healthy controls	miRNA-93 (Up)	-	-	[102]
Squamous cell carcinoma of the head and neck	522 SCCHN samples + 44 healthy controls	miRNA‐93‐5p (Up)	Upregulation in SCCHN is associated with a poor prognosis	Metastasis of lymph nodes	[99]
Renal cell carcinoma	138 paired ccRCC sample	miRNA-93-3p (Up)	Upregulation is correlated with poor prognosis	-	[93]
Endometrial cancer	100 EC patient	miRNA-93 (Up)	Upregulation is associated with Poor overall median survival	Lymph node involvement	[103]
Retinoblastoma	23 human RB + 12 normal retinae	miRNA-93-5p (Up)	-	-	[95]
Osteosarcoma	-	miRNA-93-3p (Downregulated (by LncRNA AWPPH))	Downregulation is associated with OS poor prognosis	TNM stage, metastasis	[96]

**Fig. 4:**
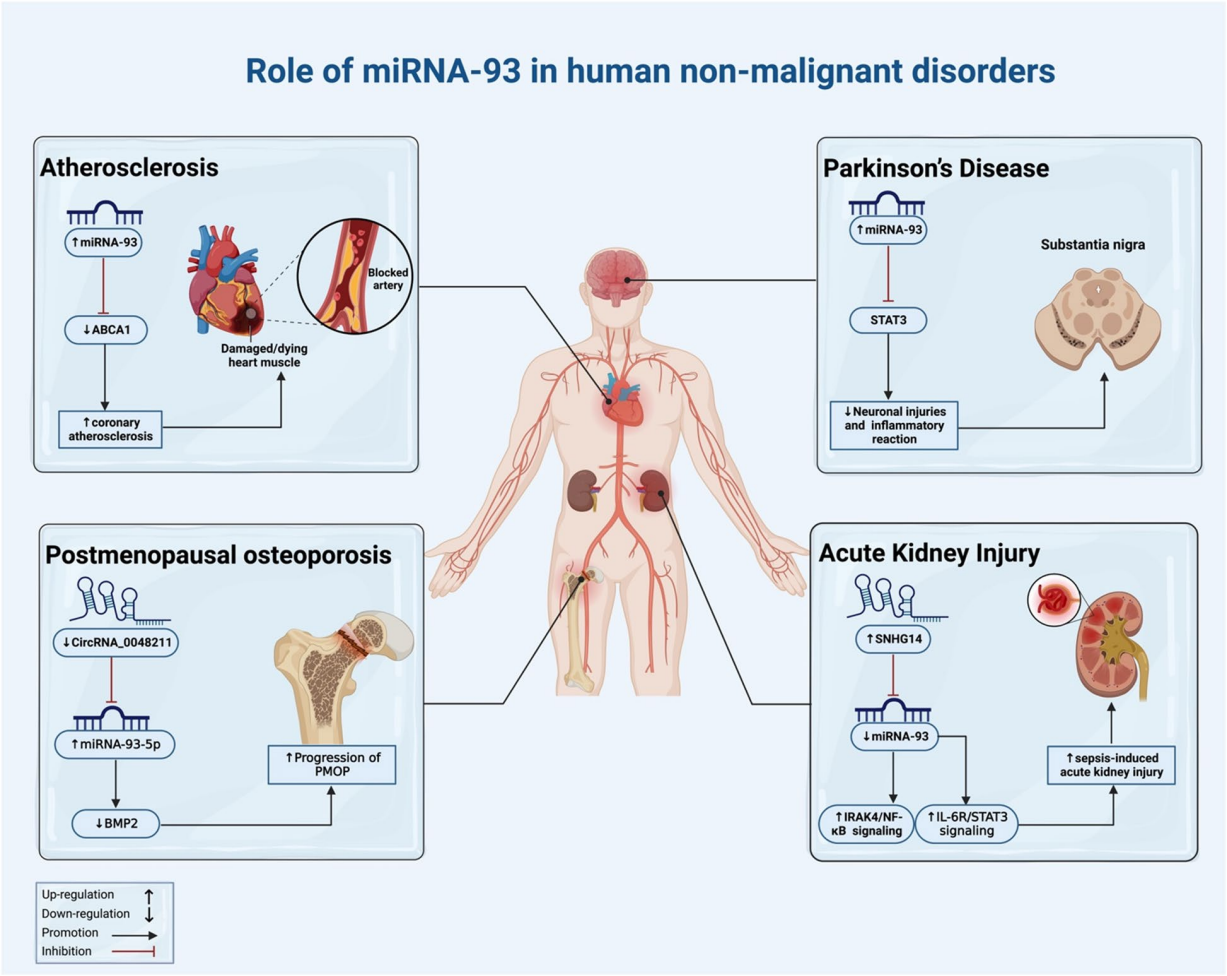
The diagram depicts the primary roles that miRNA-93 plays in the pathophysiology of non-cancerous illnesses. The expression level of miRNA-93, which modulates a large number of signaling pathways, has a role in the progression of a wide variety of diseases

Numerous studies have used cell lines, animal models, and human clinical data to study the role of miRNA-93 in human disease (Fig. 4). Based on the data from three main sources, we outlined miRNA-93's function in human disorders in the sections below.

### miRNA-93 in human disorder cell lines

miRNA-93 is showing to be one of the most crucial regulators of gene expression, and studies have demonstrated that its dysregulation has a role in a variety of diseases (Table 4). For instance, Shi et al. found that SNHG14 is increased in LPS-induced HK-2 cells and that sepsis accelerates the cellular injury of AKI caused by IL-1β, LPS, and IL-6 [104]. As a result, the *IRAK4/NF-B*, *IL-6R/STAT3*, and miRNA-93 signaling pathways may be activated by SNHG14 via miRNA-93 as a potential method [104]. Similarly, Liu et al.’s study revealed that exosomal miRNA-93-5p inhibits toll like receptor 4 (TLR4)-mediated inflammation and Atg7-mediated autophagy to prevent myocardial injury, according to in vitro and in vivo research [105]. Further, in individuals with coronary atherosclerosis, up-regulated serum miR-93 is positively associated with raising serum cholesterol levels through targeting ATP binding cassette subfamily A member 1 (*ABCA1*) [23]. These data suggest that miRNA-93 dysregulation is one of the most significant variables influencing the development of human diseases.

**Table 4:** Based on the results of cell line investigations, it can be seen how miRNA-93 contributes to the pathogenesis of diseases

Disease type	miRNA type	Interactions	Cell lines	Associated phenotype with dysregulation of miRNA-93	Ref
Acute myocardial infraction	miRNA-93-5p	TLR4/NF-κB-Mediated Inflammatory Response	H9c2	↑miRNA-93-5p, ↓Atg7-mediated autophagy and TLR4-mediated inflammatory: ↓myocardial injury	[105]
Acute kidney injury	miRNA-93	IRAK4/NF-κB and IL-6R/STAT3	HK-2	↑SNHG14, ↓ miRNA-93, ↑IRAK4/NF-κB and IL-6R/STAT3 signaling: ↑Sepsis-Induced Acute Kidney Injury	[104]
Atherosclerosis	miRNA-93	ABCA1	THP1	↑miRNA-93, ↓ ABCA1: ↑ disease progression	

### Animal studies

To assess the effect of miRNA-93 dysregulation on the development of disease, numerous animal experiments have been carried out. For instance, according to Wang et al., mice's substantia nigra lost less tyrosine hydroxylase in 13 (PD) because miRNA-93 expression was higher. This decreased the production of *STAT3*, the activation of microglia, and the inflammatory response caused by MPTP [25]. Consequently, miRNA-93 promotes Parkinson's disease through regulating *STAT3* expression. Accordingly, Xiong et al. showed that rat hepatic I-R injury is associated with both *STAT3* up-regulation and miRNA-93 down-regulation. By overexpressing miRNA-93, which also reduced inflammation and enhanced liver function, the expression of *STAT3* in rat liver I-R damage was significantly reduced [106]. Moreover, based on the Wu et al. study, miRNA-93 plays a significant role in regulating the cytotoxic effects of CAB, which can increase prolactinoma drug resistance by specifically targeting autophagy-related genes and decreasing autophagy related 7 (*ATG7*). They proved that pituitary cancers' drug resistance to CAB can be decreased by upregulating *ATG7* or silencing miRNA-93 expression in vivo xenograft models in nude mice [107]. Additionally, Yang and his team demonstrated that miRNA-93-5p expression was increased in diabetic nephropathy following the downregulation of the lncRNA XIST, which prevented the production of CDKN1A and renal interstitial fibrosis [108]. Based on these data, lncRNA XIST has been proposed as a novel prognostic biomarker and potential therapeutic target for people with DN. Table 5 provides additional details on the function of miRNA-93 in disease models in animals.

**Table 5:** Studies performed on animals to investigate the function of miRNA-93 in non-cancerous disease

Disease type	micRNA type	Animal model	Result	Ref
Parkinson’s Disease	miRNA‑93	A mouse model of PD induced by 1-methyl-4-phenyl-1, 2, 3, 6-tetrahydropyridine (MPTP)	By controlling STAT3 expression in the MPTP-induced PD mouse model, miRNA-93 lessens neuronal damage and decreases inflammatory response	[25]
Hepatic injury	miRNA-93	Rat I-R hepatic injury model	MiRNA-93 inhibits STAT3, to reduce hepatic damage during ischemia–reperfusion	[106]
Prolactinoma	miRNA-93	Rat, Female athymic nude mice	MicroRNA-93 targets ATG7 in prolactinoma to mediate cabergoline resistance	[107]
Diabetic nephropathy	miRNA-93-5p	A total of 48 C57BL/6 mice (age: 6–8 weeks, weight: 20–24 g) were used	The prevention of renal interstitial fibrosis in DN was enhanced by silenced XIST causing miRNA-93-5p-dependent CDKN1A suppression, suggesting a potential future method for DN progression prevention	[108]

### Studies in clinical samples

According to evidence from recent studies, the expression of miRNA-93 varies among various human datasets. Research on these samples shows that changes in miRNA-93 expression are associated either positively or negatively with its target genes (Table 6). For instance, hsa-miRNA-93-5p expression and *MMP-3* promoter methylation were found to be potential biomarkers for the etiology of RA and the development of the disease, as reported by Celik et al. [22]. Likewise, Qiao et al. found that miRNA-93-5p is negatively targeted by circRNA 0,048,211 in order to upregulate BMP2, which increases the progression of postmenopausal osteoporosis [109]. Similarly, in peripheral arterial disease, Shu et al. demonstrated that miRNA-93 promotes endothelial cell proliferation, migration, and tube formation, which is linked to decreased *CDKN1A* expression and is a factor in angiogenesis [110]. Moreover, Qiao et al. investigated that circRNA_0048211 was overexpressed, which increased RUNX family transcription factor 2 (*RUNX2*), osteopontin (OPN), and osteocalcin (OCN) and increased ALP activity. MiRNA-93-5p has a direct target in *BMP2* and could be sponged by CircRNA 0,048,211. As a result, circRNA 0,048,211 prevents postmenopausal osteoporosis [109]. Furthermore, Chen et al. revealed that, through direct targeting of the *GLUT4 3' UTR* in adipocytes, miRNA-93 overexpression caused glucose transporter type 4 (*GLUT4*) gene expression to be downregulated, whereas miRNA-93 activity inhibition caused *GLUT4* expression to be upregulated. These findings show that miRNA-93 expression is elevated in all PCOS patients as well as in non-PCOS women who have IR, potentially explaining the IR of the disease [111]. Although, they suggest a unique method for controlling insulin-stimulated glucose uptake via miRNA-93.

**Table 6:** Studies of clinical samples investigate the effect of miRNA-93 in diseases that are not malignant

Disease type	miRNA type	Number of clinical samples	Targets/pathways	Expression	Function	Ref
Polycystic ovary syndrome	miRNA-93	25- PCOS and biochemical hyperandrogenemia	-	Upregulated	MiRNA-93 levels were higher in the blood of PCOS patients who had higher insulin and testosterone levels	[112]
	miRNA-93	41 subjects (20 control and 21 PCOS)	GLUT4	Upregulated	In PCOS-associated adipose tissue (AT), overexpression of miRNA-93 decreases GLUT4 expression	[111]
Postmenopausal osteoporosis	miRNA-93-5p	Patients with PMOP (*n* = 30) and controls (*n* = 30)	CircRNA_0048211/ miRNA-93- 5p/BMP2	Downregulated (by circRNA_0048211)	MiRNA-93-5p is negatively targeted by CircRNA 004,211, which increases BMP2 and slows the course of PMOP	[109]
Peripheral arterial disease	miRNA-93	146 sample with PAD (79 male and 67 female)	CDKN1A	Upregulated	MiRNA-93 increases angiogenesis by increasing EA. hy926 endothelial cell proliferation, migration, and tube formation, which decreases CDKN1A expression	[110]
Mild Head Trauma	miRNA 93	59 sample and 91 controls	-	Upregulated	Validity of Serum miRNA 93 Can Reduce the Need for CT scans in Patients with Mild Head Injury	[113]
Multiple blunt trauma	miRNA-93	A total of 60 healthy controls and 50 consecutive persons with MBT who are matched for age and sex	-	Upregulated	In individuals with multiple traumas, miRNA-93 may be a helpful biomarker for assessing the severity of the injuries	[114]
Periodontitis	miRNA-93	3 sample	HIF-1α, NFAT5	Upregulated	MiRNA-93 expression increased in periodontitis patients, whereas NFAT5 mRNA expression decreased. Additionally, hypoxic environments cause GMSCs to increase HIF-1 expression	[115]
Chronic kidney disease	miRNA-93-5p	67 CKD patients with KT, 73 patients with CKD stages 3 to 5, and 36 healthy controls	-	Downregulated	Levels of miRNA-93-5p are linked to CKD stage, inflammation, and bone metrics	[116]
Rheumatoid arthritis	miRNA-93-5p	49 RA sample and 38 controls	MMP-3, IL-16	Upregulated	MMP-3 promoter methylation and miRNA-93-5p expression levels may serve as helpful biomarkers for the pathophysiology of RA	[22]

## miRNA-93 related signaling pathways in human disorders

miRNA-93 can target different signaling pathways and induce the progression of the vast majority of malignancies [117]. Wu et al. showed that miRNA-93-5p produces drug resistance in pancreatic cancer (PCa) cells and promotes cancer growth using the *PTEN*-mediated *PI3K/Akt* signaling pathway [30]. Furthermore, the *AKT/mTOR/VEGF* pathway might be regulated by miR-93 in AML. For example, it was reported that mTOR is involved in AML's tumor-associated angiogenesis, vascular endothelial growth factor (VEGF) production, and leukemic cell proliferation [118]. The molecular analysis showed that miR-93 was found to suppress AKT's phosphorylation [119]. Additionally, miR-93 induced *PI3K/AKT* signaling, which facilitated the proliferation, invasion, and metastasis of cancer cells [120, 121]. The *miR-93/PTEN/AKT* signaling pathway has been linked to drug resistance in cancer cells when miR-93 is overexpressed [122]. AML cells also have an increased *PI3K-Akt-mTOR* signaling pathway, which eventually contributes to the metabolic remodeling of AML [123].

Additionally, in cancer cells, miRNA-93 enhances *TGF-β* and induces epithelial-to-mesenchymal transformation through specific gene targeting. QU et al. showed that miRNA-93 overexpression in LC cells facilitated *TGF*-induced EMT by suppressing *NEDD4L* [124]. Reducing *NEDD4L* improves *TGF*- signal transduction and promotes *TGF-β* induced EMT by protecting activated *SMAD2/SMAD3* from degradation [124]. Likewise, by stimulating the Hippo pathway, miRNA-93-5p improves the proliferative, migratory, and invasive properties of GC cells. The Hippo pathway could be inhibited by miRNA-93-5p overexpression, whereas miRNA-93-5p knockdown may potentially promote Hippo signaling [125]. The protein levels of Hippo pathway regulators, protocadherin fat 4 (*AKA*, cadherin family member 14 (*CDHF14*)), and Large Tumor Suppressor Kinase 2 (LATS2) have also been shown to be repressed by the upregulation of miRNA-93-5p, which may be used as a diagnostic and therapeutic target for GC [125].

On the other hand, miRNA-93 inhibits the progression of a number of malignancies by impeding some signaling pathways, such as the *Wnt/β-catenin* pathway. For instance, using an experiment that measures the expression of β-catenin, axin, c-Myc, and cyclin-D1, Tang and his colleagues found evidence that miRNA-93 may downregulate the *Wnt/β-catenin* pathway in CRC cells [126].

Moreover, in vitro miRNA-93 overexpression greatly reduced the ability of BC cells to invade and proliferate in 3D organoids, and it reduced their capacity to spread to the liver in vivo [127].

Furthermore, Shang et al. found that through the *TLR4/NF-β* signaling pathway, miRNA-93 controls neurological function, cerebral edema, and neuronal death in rats with intracerebral hemorrhage [128].

In breast tumors, miRNA-93 acts as a metastasis inhibitor by repressing invasion and stem cell characteristics. Shibuya et al. found that the protein level of *WASF3* in BC cells was decreased by miR-93 overexpression, and *WASF3* restored the miR-93-mediated inhibition of BC development [129].

Taken together, these studies suggest that miRNA-93 is responsible for regulating signaling pathways in the tumor growth of human malignancies, and it can be used as a possible therapeutic target.

## MiRNA-93 and drug resistance

Drug resistance remains a significant health concern that restricts the effectiveness of cancer chemotherapy [130] and is a significant issue in the care of cancer patients. miRNAs play a significant role in tumor growth and therapeutic resistance [131]. Further, cancer chemotherapeutic resistance is a major challenge to the fight cancer disease. According to the statistics, over 90% of cancer patient mortality is associated with drug resistance [132]. Cancer drug resistance can be developed by numerous factors, including reduced anticancer drug absorption, changed drug targets, altered cell cycle checkpoints, and enhanced DNA damage repair. Many researches have revealed that miRNAs target drug-resistance genes or influence cell growth, and apoptosis genes to make cancer cell therapy resistant.

One of these, miRNA-93, is essential for the development of drug resistance in a variety of malignancies by interacting either with coding genes [133] or non-coding genes [40], as well as by altering the targeted pathways [134]. For instance, Wu et al. revealed that miRNA-93-5p induces resistance to gemcitabine via targeting the *PTEN*-mediated *PI3K/Akt* signaling pathway in pancreatic cancer (PCa) cells [30]. Likewise, miRNA-93 promotes cabergoline resistance in prolactinoma through targeting *ATG7* [107]. Further, lncRNA CTBP1-AS2 induces the activation of the *TGF-β/SMAD2/3* pathway via inhibiting miR-93-5p, thereby accelerating the development of CRC [40]. Similarly, upregulated miRNA-93 induces cisplatin-resistant ovarian cancer cells through directly targeting *PTEN*, which in turn co-regulates the *PTEN/Akt* signaling pathway [131]. Additionally, tumor initiating cells (T-ICs) play an important role in tumor development, metastasis, recurrence, and drug resistance in liver cancer [135]. Li et al. demonstrated that miRNA-93 was significantly upregulated and regulated liver T-ICs by binding to the 3′-UTR of myotubularin-related protein 3 (*MTMR3*) in cisplatin- or sorafenib-resistant liver cancer tissues [56]. Furthermore, it has been demonstrated that miRNA-93 plays a role in the development of MDR in prolactinoma [136] and ovarian cancer cells [131]. Interestingly, Hu et al. showed that elevated miRNA-93 expression is correlated with BC resistance, and they proved that miRNA-93 expression is controlled by DNA demethylation [137].

**Fig. 5:**
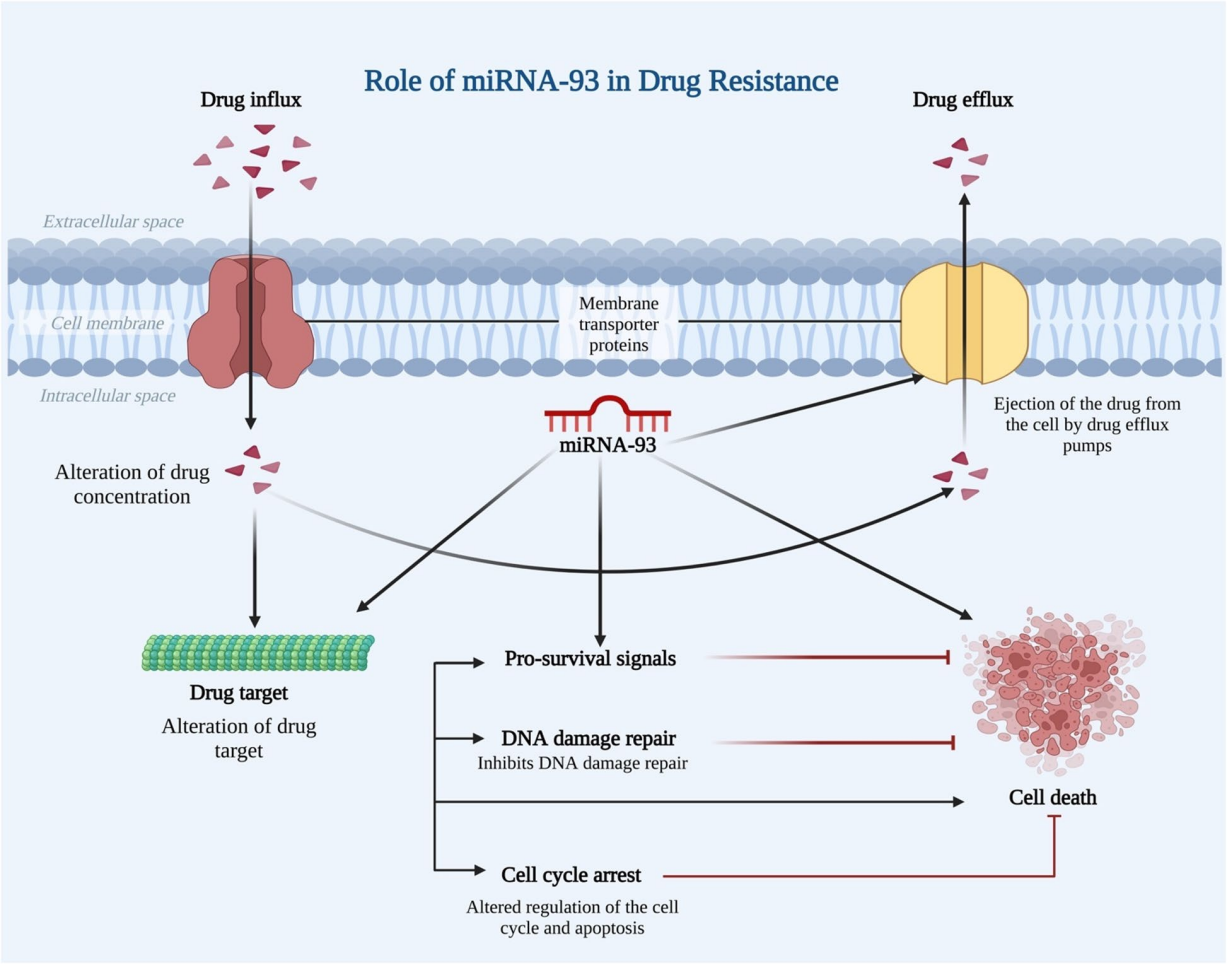
Illustration shows the role of miRNA-93 in drug resistance through different mechanisms such as alteration of drug concentration, drug target, cell cycle and apoptosis, and inhibition of DNA damage repair mechanisms

According to the above data, miRNA-93 plays a significant role in driving therapeutic resistance in various cancer types; nevertheless, additional research will be needed to understand the mechanisms that miRNA-93 in cancer patients triggers to resist chemotherapy (Fig. 5).

## Discussion

Recent studies indicate that miRNAs have significant roles in human disorders as well as the initiation, development, and metastasis of cancer, which are expressed abnormally [138, 139]. Multiple cancers have abnormal miRNA-93 levels, and their expression levels are associated with a poor prognosis [60]. Furthermore, miRNA-93 is significantly dysregulated in chemo-resistant cell lines, animal models, and clinical tumor samples.

Several studies revealed that upregulated levels of lncRNAs and circRNAs can target miRNA-93 or specific parts of this miRNA. For example miRNA-93 has been found to be sponged by some lncRNAs and circRNAs, namely lncRNA PTENP1 [140], lncRNA H19 [48], lncRNA-XIST [141], LINC01116 [142], lncRNA MEG3 [143], LINC00472 [144], lncRNA SNHG14 [145], lncRNA AWPPH [96], lncRNA CA3-AS1 [146], lncRNA ASMTL-AS1 [89], lncRNA ZNF667‐AS1 [147], and lncRNA SNHG16 [148], circRNF13 [149], cESRP1 [150], circRNA VPRBP [151]. These findings highlighted the complexity of the network that miRNA-93 uses to carry out its actions. Along with chromosomal polymorphisms at the miRNA-93 gene locus, it is thought that abnormal up-regulation of the circRNAs or lncRNAs could cause miRNA-93 to be turned down. As a result, the up-regulation of lncRNAs and circRNAs that sponge miRNA-93 is a well-known mechanism for its downregulation in many cancers. Although loss in the genomic region that codes for miRNA-93 is a putative explanation, the mechanism behind miRNA-93's down-regulation in malignant tissues is not fully understood.

Furthermore, the link between miRNA-93 expression levels and patient outcomes shows how this miRNA could be used as a biomarker to predict how well a cancer patient will do. However, miRNA-93 performs diverse roles in different cancers; therefore, the patterns and orientations of these relationships depend on the roles that miRNA-93 plays in each cancer type.

Additionally, miRNA-93 plays a crucial role in the pathophysiology of illnesses that are not cancerous, such as atherosclerosis, hepatic injury, diabetic nephropathy, rheumatoid arthritis, prolactinoma, osteoarthritis, Parkinson’s disease, rheumatoid arthritis, and acute myocardial infarction. However, the diagnostic use of this miRNA is complicated by the dysregulation of miRNA-93 in cancerous and non- cancerous diseases originating from a particular tissue.

Meanwhile, the best explanation for how this miRNA contributes to the pathophysiology of both malignant and non-malignant illnesses is provided by its substantial role in the regulation of signaling pathways that control cell proliferation and death.

## Conclusions

miRNA-93 is an example of a miRNA having tissue-specific effects on cancer development. miRNA-93 may be useful in the clinical diagnosis and prognosis of cancer, which play a role in the progression of malignancy and chemotherapy resistance. Its role in this process depends on the type of tissue, because it can help cancer cells grow in some tissues and stop them in others. However, studies on miRNA-93 have been conducted in cell lines, animal models, and clinical samples in malignant and non-malignant conditions. In cell line studies of malignant condition, miRNA-93 has been shown to regulate various cellular processes such as cell proliferation, migration, and apoptosis. Although, animal studies have also explored the role of miRNA-93 dysregulation in different cancers by using different animal models. In clinical samples, miRNA-93 has been found to be dysregulated in various cancers and may serve as a potential biomarker for diagnosis and prognosis. In cancer cell lines and animal models of the disease, targeting miRNA-93 has been shown to be a practical and effective way to stop cancer cells from spreading and reduce the size of tumors. In non-malignant conditions, miRNA-93 has also been shown to play a role in various diseases based on cell line, animal, and clinical studies. Further, studies on miRNA-93 suggested that it plays a role in various cellular processes and may be a promising target for the development of novel therapies in both malignant and non-malignant conditions. The viability of these strategies in a clinical environment has not yet been assessed. To expand new insights into this area, additional study is required.

## Data Availability

The analyzed data sets generated during the study are available from the corresponding author on reasonable request.
